# The anatomical features of the lateral femoral cutaneous nerve with total hip arthroplasty: a comparative study of direct anterior and anterolateral supine approaches

**DOI:** 10.1186/s12891-022-05224-w

**Published:** 2022-03-18

**Authors:** Taku Ukai, Kaori Suyama, Shogo Hayashi, Haruka Omura, Masahiko Watanabe

**Affiliations:** 1 Department of Orthopedic Surgery, Surgical Science, Tokai University School of Medicine, 143 Shimokasuya, Isehara, Kanagawa 259-1193 Japan; 2 Department of Anatomy, Division of Basic Medical Science, Tokai University School of Medicine, Isehara, Kanagawa 259-1193 Japan

**Keywords:** Lateral femoral cutaneous nerve, Direct anterior approach, Anterolateral supine approach

## Abstract

**Background:**

Lateral femoral cutaneous nerve (LFCN) injury after total hip arthroplasty causes patient dissatisfaction. This cadaveric study aimed to assess the risk for LFCN injury after the direct anterior approach (DAA) and anterolateral supine approach (ALS) with a focus on the anatomical variations of the LFCN.

**Methods:**

Thirty-seven hemipelves from 20 formalin-preserved cadavers (10 males and 10 females) were dissected to identify the LFCN, evaluate variations, and measure the distance from the LFCN to each approach. The LFCN was classified as classical, late, multi trunk, or primary femoral.

**Results:**

There were no significant variations in the LFCN between the sexes. The distance from the LFCN to DAA incision (10 [0–17.8] mm) was significantly less than that from the LFCN to ALS incision (27 [0–40] mm); moreover, 64.9% of DAA incisions crossed the LFCN. The classical type LFCN was closest to the DAA incision. The DAA incision most frequently crossed the LFCN at the proximal third, and the frequency of intersection of the LFCN and DAA incisions decreased by 25% by a 10-mm shortening of the DAA proximal incision. In contrast, 27% of ALS incisions crossed the LFCN. Multi trunk type LFCN was closest to the ALS incision. There were no significant differences between each approach and LFCN variations, and the frequency of intersection of the LFCN and ALS incisions decreased by 20% by a 10-mm shortening of the ALS proximal incision.

**Conclusions:**

The intersection rates between the LFCN and the DAA and between the LFCN and the ALS were approximately 65 and 30%, respectively. Approximately 20–25% of these injuries may be avoidable by a 10-mm shortening of the proximal incision.

## Background

Various approaches are used for total hip arthroplasty (THA) [1–3]. Among them, the anterior approaches have been used recently because these approaches take advantage of the intermuscular plane [4–6]. Especially, these approaches have the advantage of low dislocation rate [7] and early muscle recovery [8]. Anterior approaches are divided into the direct anterior approach (DAA) and the anterolateral supine approach (ALS). The DAA is modified from the Smith–Peterson approach that takes advantage of the intermuscular plane between the sartorius and tensor fascia latae. Although this approach does not involve the need to cut any muscles, some studies have reported that 23.3–30% of patients who underwent THA via the DAA experienced numbness in the lateral thigh owing to injury of the lateral femoral cutaneous nerve (LFCN) [9, 10]. The ALS approach is also an intermuscular approach that invades between the tensor facia latae and the gluteus medius. However, the rate of gluteal insufficiency after the ALS is higher than that after the DAA [11]. Similar to the DAA, this approach does not require muscle resection that can cause dislocation after THA. Additionally, this approach can preserve the iliofemoral ligament and anterior capsule of the hip. Preserving these soft tissues is useful not only for stability but also for preventing leg discrepancy and excess lateral offset. Compared with the DAA, the ALS rarely causes LFCN injury, but the accurate percentage of LFCN injury in the ALS remains unclear.

The LFCN is derived from the second and third lumbar nerves. After running through the medial side of the anterior superior iliac spine (ASIS), the nerve runs on the sartorius and tensor fasciae latae before reaching the anterolateral region of the thigh. The LFCN does not have motor functions; it is a purely sensory nerve. Thus, even if the nerve is injured during an operation, major complications, such as sciatica and nerve palsy, are not observed. However, some patients have numbness and hypoesthesia of the lateral thigh due to LFCN injury. The numbness and pain can become a “meralgia paresthetica,” which is a chronic pain syndrome with very unpleasant dysesthesias. It has been reported that LFCN injury causes numbness of the lateral thigh leading to patient dissatisfaction [12, 13]. Although the LFCN has several anatomical variations, only few reports have investigated the impact of LFCN variation on the surgical approach [14, 15]. The purpose of this cadaveric study was to assess the risk for LFCN injury after the DAA and ALS by evaluation of anatomic landmarks.

## Methods

**Fig. 1:**
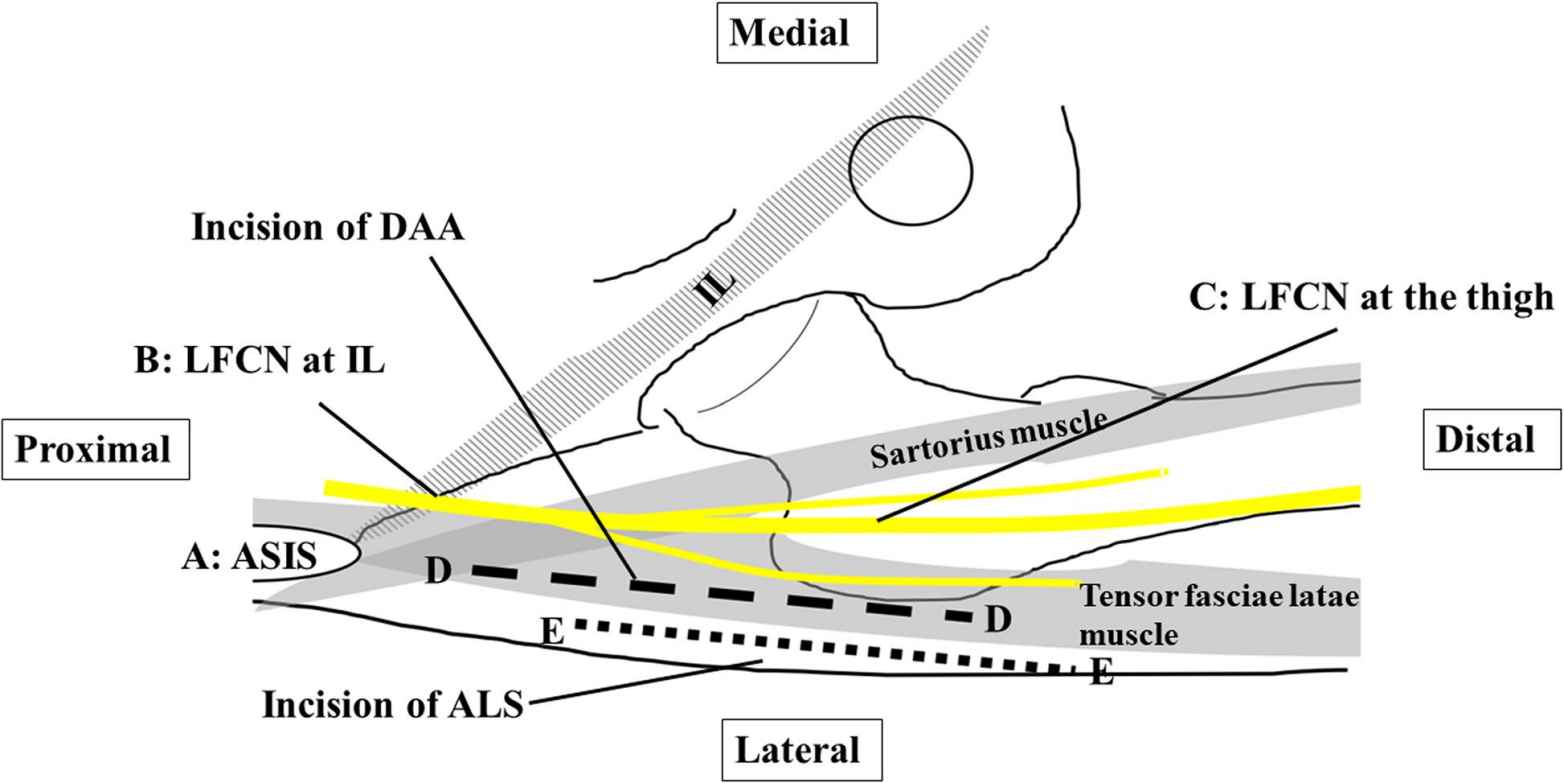
Schematic drawing of marked points. Point A represents the ASIS. Point B represents the LFCN at the level of IL. Point C represents the LFCN at the level of the thigh. Point D represents the skin incision of the DAA. Point E represents the skin incision of the ALS. ASIS, anterior superior iliac spine; ALS, anterolateral supine; DAA, direct anterior approach; IL, inguinal ligament; LFCN, lateral femoral cutaneous nerve

All cadaveric studies were performed at the University of Tokai in Isehara, Japan. In total, 37 hemipelves from 20 formalin-preserved cadavers (10 male and 10 female) with a mean age of 75.3 (range, 62–99) years were dissected. The LFCN was identified at the ASIS level by cutting the inguinal ligament (IL), and the LFCN was traced from the ASIS to the lateral thigh. Pins were placed at 5 points (A: ASIS, B: LFCN at the level of the IL, C: LFCN at the level of the thigh, D: skin incision of the DAA, E: skin incision of the ALS) (Fig. 1). Two observers performed all measurements. A 100-mm skin incision for the DAA was marked from a point 20 mm lateral and 20 mm distal to the ASIS to a point 20 mm lateral to the head of the fibula [14]. A 100-mm skin incision for the ALS was marked at the anterior border of the trochanter and 6 cm caudal and 4 cm cranial to the trochanter tip. The distances between the LFCN and skin incisions of the DAA (C–D) and ALS (C–E) were measured based on a previous report [14]. We measured the distance from the most proximal point of the incision to the point at which it crossed the LFCN. When the incision did not cross the LFCN, we measured the minimum distance between the LFCN and the incision.

**Fig. 2:**
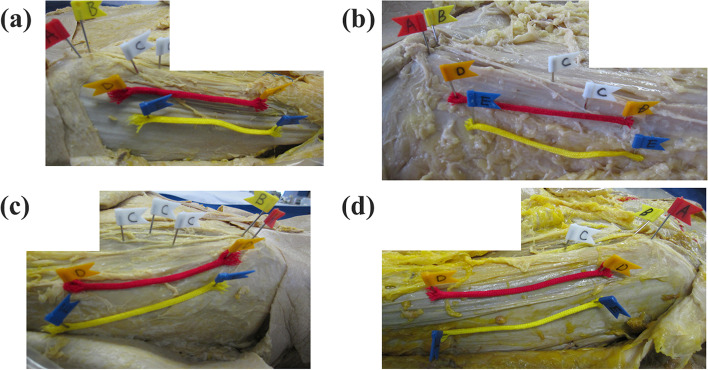
Variations in the LFCN. **a** Classical-type LFCN. The classical type is identified as branches of the LFCN that divide at the level of the IL. **b** Late-type LFCN. The late type is identified as branches of the LFCN that divide after running through the IL. **c** Multi trunk-type LFCN. Multi trunk type is identified as several equally sized main nerve trunks. **d** Primary femoral-type LFCN. The primary femoral type is identified by the lack of an evident branch from the main trunk of the LFCN. Points A (red flags), B (yellow flags), C (white flags), D (orange flags), and E (blue flags) represent the ASIS, the LFCN at the level of the IL, the LFCN at the level of the thigh, the edge of the skin incision of the DAA (red lines represent the skin incision of the DAA), and the edge of the skin incision of the ALS (yellow lines represent skin incision of the ALS). ASIS, anterior superior iliac spine; ALS, anterolateral supine; DAA, direct anterior approach; IL, inguinal ligament; LFCN, lateral femoral cutaneous nerve

We divided the LFCN into four types (classical type: gives rise to a distinct main tract and gluteal branches around the level of the IL; late type: a main tract and other branches after passing beyond the upper thigh; multi trunk: the LFCN gives rise to several equally-sized main branches that travel across the thigh; primary femoral: no evident branch that runs on the anterolateral thigh (Fig. 2) [16]. We identified the main branch as a main tract or a branch with a diameter more than half of the diameter of the main tract at the IL level; similarly, the small branches were identified as those with diameters less than half of the diameter of the main tract at the IL level.

### Statistical analyses

The Wilcoxon signed-rank sum test was used to compare the distance from the DAA and ALS incisions to the LFCN between the sexes as well as the distance from each approach to the LFCN. The chi-squared test and residual analysis were used to evaluate the LFCN patterns between the sexes, the frequency of intersection of each approach and LFCN variations, and the point at which the LFCN is crossed by each incision. The level of significance was set at *p* < 0.05. Analyses were performed using the SPSS statistical software version 26 (IBM Corp., Armonk, NY, USA).

## Results

**Fig. 3:**
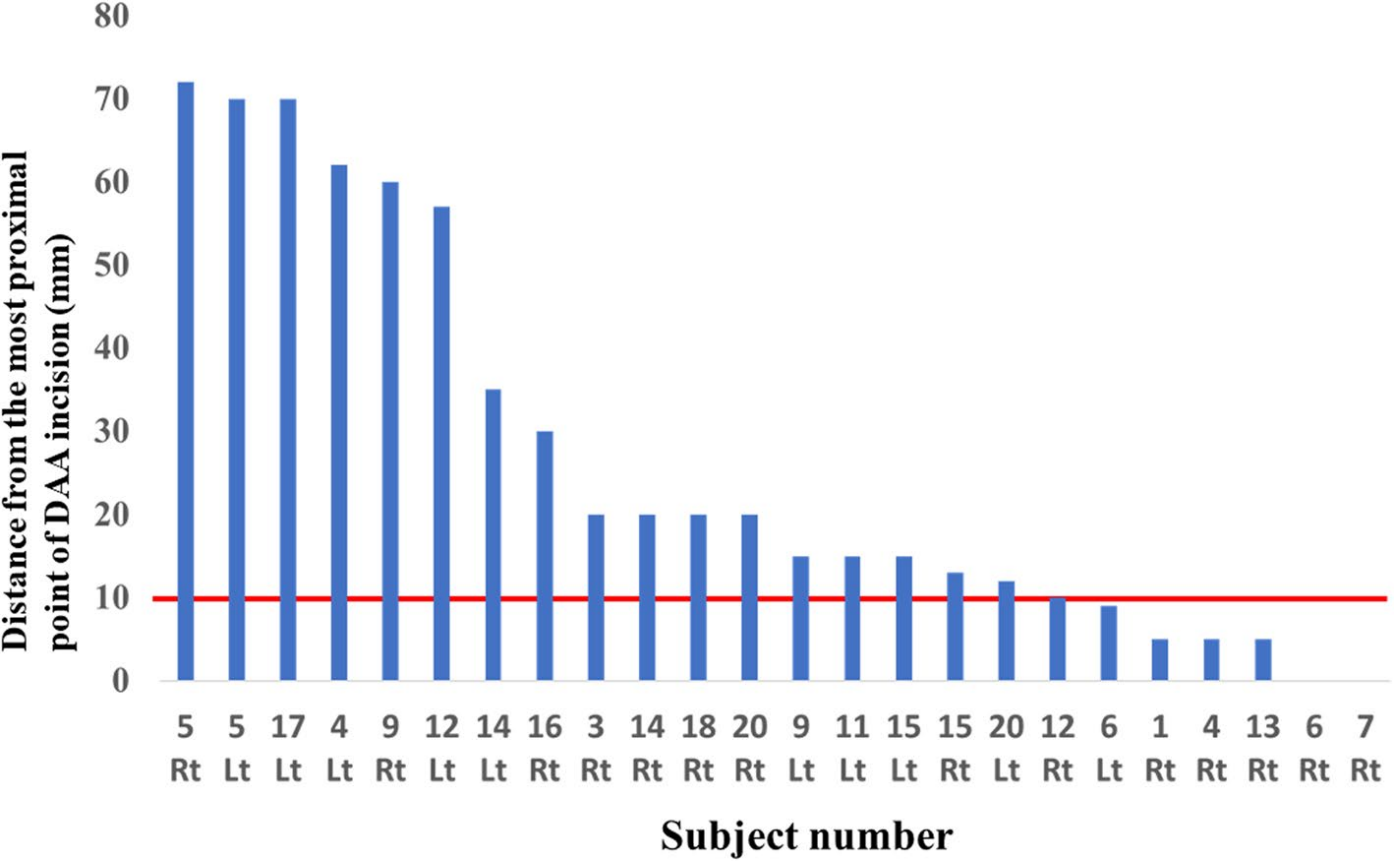
The intersection point of the LFCN and DAA incision. Blue bars represent the distance from the most proximal point of the DAA incision to the LFCN for each subject. DAA, direct anterior approach; LFCN, lateral femoral cutaneous nerve; Lt, left hip; Rt, right hip

The variations in the LFCN were as follows: classical, 19 hips (51.4%); late, 7 hips (18.9%); multi-trunk, 6 hips (16.2%); and primary femoral, 5 hips (13.5%). The LFCN variations in men were as follows: classical, 10 hips; late, 5 hips; multi-trunk, 4 hips; and primary femoral, 1 hip. The LFCN variations in women were as follows: classical, 9 hips; late, 2 hips; multi-trunk, 2 hips; and primary femoral, 4 hips. The LFCN variations were not significantly different between the sexes. The distance between the LFCN and the DAA (C–D) and ALS incisions (C–E) were 10 [0–17.8] mm and 27 [0–40] mm, respectively. The DAA incision was significantly closer to the LFCN than the ALS incision (95% confidence interval, − 22.1– − 7.6, *p* < 0.001). In 24 out of 37 hips (64.9%), the DAA incision crossed the LFCN. The DAA incision crossed 13 and 11 main and small branches of the LFCN, respectively. Only the DAA approach was found to cross the LFCN in 14 hips. Both approaches crossed the LFCN in 10 hips. Neither approach crossed the LFCN in 13 hips. The classical type was the closest to the DAA incision (2.6 ± 7.8 mm), followed in order by the multi trunk type (5 ± 12.2 mm), late type (12.4 ± 10.3 mm), and primary femoral type (21.8 ± 4.3 mm) (Table 1). Concerning variations in the LFCN, the classical type was crossed by the DAA incision most frequently (Table 2). In contrast, the late and primary femoral variations were crossed by the DAA incision less frequently than other variations (Table 2). In 17, 4, and 3 hips, the incision crossed the LFCN at the proximal third, middle third, and distal third, respectively (Table 3). Specifically, in 12 out of 17 classical-type LFCNs, the DAA incision crossed the nerve at the proximal third area (Table 3). Additionally, the intersection rate of the LFCN and DAA incisions decreased by 25% by a 10-mm shortening of the DAA proximal incision (Fig. 3).

**Table 1:** Distance from each incision to LFCN variations

	DAA	ALS
Classical (mm)	2.6 ± 7.8	15.6 ± 17.9
Multi trunk (mm)	5 ± 12.2	15 ± 17.6
Late (mm)	12.4 ± 10.3	34 ± 16.7
Primary femoral (mm)	21.8 ± 4.3	40 ± 6.1

*ALS* Anterolateral supine, *DAA* Direct anterior approach, *LFCN* Lateral femoral cutaneous nerve

**Table 2:** Frequency of intersection of the DAA incision and LFCN variations

	Cross with the LFCN	No cross with the LFCN	*p* < 0.001^a^
Classical	17	2	
Adjusted residual	3.2	−3.2	
Late	2	5	
Adjusted residual	−2.2	2.2	
Multi trunk	5	1	
Adjusted residual	1.0	−1.0	
Primary femoral	0	5	
Adjusted residual	−3.3	3.3	

*DAA* Direct anterior approach, *LFCN* Lateral femoral cutaneous nerve

^a^ A chi-square test and residual analysis were performed between LFCN injury of the DAA incision and LFCN variations. The level of significance was set at *p* < 0.05

**Table 3:** Intersection points of the LFCN crossing with the DAA incision

	Proximal	Middle	Distal	*p* = 0.49
Classical	12	3	2	
Late	1	0	1	
Multi trunk	4	1	0	

*DAA* Direct anterior approach, *LFCN* Lateral femoral cutaneous nerve

^a^A chi-square test was performed between the intersection point of the DAA incision and the LFCN variations. The level of significance was set at *p* < 0.05

**Fig. 4:**
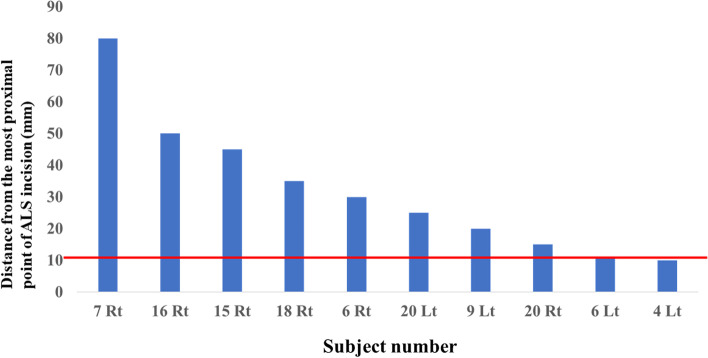
The intersection point of the LFCN and ALS incision. Blue bars represent the distance from the most proximal point of the ALS incision to the LFCN for each subject. ALS, anterolateral supine approach; LFCN, lateral femoral cutaneous nerve; Lt, left hip; Rt, right hip

In 10 out of 37 hips (27%), the ALS incision crossed the LFCN. The ALS incision crossed five main branches and five small branches of the LFCN. The multi-trunk type was the closest to the ALS incision (15 ± 17.6 mm), followed in order by the classical type (15.6 ± 17.9 mm), late type (34 ± 16.7 mm), and primary femoral type (40 ± 6.1 mm) (Table 1). Concerning variations in the LFCN, there was no significant difference between the frequency of intersection of the ALS incision and LFCN variations (Table 4). Additionally, in six, three, and one hip, the incision crossed the LFCN at the proximal third, middle third, and distal third, respectively (Table 5). Specifically, in five out of the seven classical-type LFCNs, the ALS incision crossed the nerve at the proximal third area (Table 5). Finally, the intersection rate of the LFCN and ALS incision decreased by 20% by a 10-mm shortening of the ALS proximal incision (Fig. 4).

**Table 4:** Frequency of intersection of the ALS incision and LFCN variations

	Cross with the LFCN	No cross with the LFCN	*p* = 0.32
Classical	7	12	
Late	1	6	
Multi trunk	2	4	
Primary femoral	0	5	

*ALS* Anterolateral supine, *LFCN* Lateral femoral cutaneous nerve

^a^A chi-square test was performed between the LFCN injury of the ALS incision and the LFCN variations. The level of significance was set at *p* < 0.05

**Table 5:** Intersection points of the LFCN crossing with the ALS incision

	Proximal	Middle	Distal	*p* = 0.04
Classical	5	2	0	
Late	0	0	1	
Multi trunk	1	1	0	

*ALS* Anterolateral supine, *LFCN* Lateral femoral cutaneous nerve

^a^ A chi-square test was performed between the intersection point of the ALS incision and the LFCN variations. The level of significance was set at *p* < 0.05

## Discussion

In our study, we found that the DAA incision was closer to the LFCN than the ALS incision, while 64.9% of the DAA incisions crossed the LFCN. In contrast, 27% of the ALS incisions crossed the LFCN. The classical-type LFCN was most frequently crossed by the DAA incision. Contrastingly, late and primary femoral types were less frequently crossed by the DAA incision. The ALS incision most frequently crossed the LFCN in the proximal third area.

Injury of the LFCN is known as a complication associated with the DAA. Even after LFCN injury, most patients do not complain of motor dysfunction and functional outcomes are not affected [17]. Although Bhargava et al. [18] reported that more than 80% of patients who experienced LFCN injury recovered from sensory deficits, LFCN injury sometimes induces meralgia paresthetica or burning sensation [19]. Moreover, severe cases require surgical treatment, such as neurolysis or neurotomy [19]. It has been reported that the frequency of LFCN injury varies from 2 to 67% [18–22]. Some authors have clinically investigated LFCN injury after the DAA meticulously. Homma et al. reported that LFCN injury was observed in 31.9% of hips [10]. Takada et al. clinically investigated the rate of LFCN injury after the DAA and ALS [9]. They reported that LFCN injury after the DAA was observed in 23.3% of cases, whereas LFCN injury was not observed after the ALS. Similar to our study, incidents of LFCN injury have been reported after performing cadaveric studies. In particular, Rudin et al. reported that 33% of LFCN injuries were unavoidable during the use of an anterior approach [14]. Bartlett et al. reported that 44% of the LFCNs crossed the anterior approach incision in their cadaveric study. A large difference was noted in the rate of LFCN injury between previous clinical studies and our cadaveric study. We believe that there are two reasons for the divergent results. First, the rate of LFCN injury varies largely in the literature (2–67%) [18–22]. Thus, the accurate LFCN injury rate remains unclear. Second, our cadaveric study revealed that 35.1% of the main branches crossed the DAA incision. This percentage was approximately consistent with the rate of clinical LFCN injury [10, 23]. Therefore, injury of the small branches of the LFCN may not be of clinical significance.

As for variations in LFCN, Bartlett et al. reported that there are four types of variations in LFCN (classical 64%, late 17.7%, multi trunk 4%, and primary femoral 13%) [16]. In our case, the proportion of the variations was similar to those reported previously. Some previous reports have classified the LFCN into two [15], three [14], or four types [16]. We believe that it is important to classify the LFCN meticulously to assess LFCN injury. Therefore, we classified the LFCN into four types in this study.

**Fig. 5:**
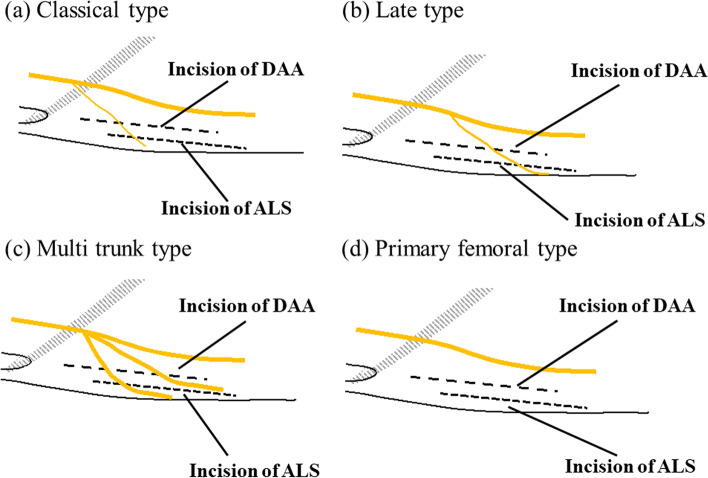
Schematic diagram of variations in the LFCN. **a** The classical type crossed the DAA and ALS incisions at the proximal third. **b** The late. Type crossed the DAA and ALS incisions at the proximal and distal thirds. **c** The multi trunk type crossed the DAA and ALS incisions at the proximal third. **d** The primary femoral type had the lowest risk for LFCN injury owing to the lack of an anterolateral branch. ALS, anterolateral supine; DAA, direct anterior approach; LFCN, lateral femoral cutaneous nerve

Among the variations in the LFCN, the classical type was the most at risk for LFCN injury (Table 2). The gluteal branch of the classical type branches off earlier than the other variations. Therefore, the femoral branch of the classical-type variant was crossed by the proximal part of the incision. For that reason, it is important to shorten the proximal incision or not to extend the proximal incision in the classical-type LFCN to reduce injury. The gluteal branch in the late-type LFCN branches off later than in the classical type. Thus, the late-type LFCN was less frequently injured than the classical type. Although the LFCN injury in the late type is low, the areas where the incision crossed the LFCN varied. Thus, predicting and avoiding late-type LFCN injuries are difficult. The number of multi trunk-type LFCNs was low in the present study; however, the multi trunk-type LFCN had the second highest rate of LFCN injury among all the LFCN variations. One of them ran laterally along the side of the thigh and was crossed by the DAA and ALS incisions. Thus, we believe that shortening the proximal incision can reduce multi trunk-type LFCN injury, especially in the DAA. The primary femoral type did not have an anterolateral branch and had the lowest risk for LFCN injury. These findings indicated that the risk of LFCN injury differs depending on the LFCN variations (Fig. 5). However, identifying the variations in the LFCN during surgery was very difficult because there were no significant variations in the LFCN between the sexes. Thus, we focused on the area where the LFCN was crossed by both incisions. Our findings indicated that shortening or not extending the proximal incision may help reduce the rate of LFCN injury.

There were certain limitations to this study. First, we did not evaluate the symptoms of LFCN injury because this was a cadaveric study. However, to our knowledge, this is the first report to evaluate the risk of LFCN injury for the DAA and ALS. Thus, we believe that this study will have a great impact on the risk of LFCN injury after the DAA and ALS. Second, we first identified the LFCN before setting the DAA and ALS incisions. This might have resulted in a bias. Third, the cadavers were embalmed using formalin. There are several types of preservation methods [23], and the fresh-frozen method is the most realistic method. However, this method has the disadvantage of the requirement of freezers, limited work time, and risk of infection. The Thiel-embalmed method results in soft and flexible cadavers. However, this method is more expensive than the formalin method and the dissection time is limited. The formalin method is widely used because of its lower cost than other methods. However, soft tissues lose their elasticity due to the use of formalin and this can affect the LFCN. Thus, in the future, we plan to perform cadaveric studies by using other preservation methods.

## Conclusion

The rates of intersection between the LFCN and the DAA and between the LFCN and the ALS are approximately 65 and 30%, respectively. Approximately 20–25% of these injuries may be avoidable by a 10-mm shortening of the proximal incision.

## Data Availability

The datasets used and/or analyzed during the current study are available from the corresponding author on reasonable request.

## Abbreviations

ALS: Anterolateral supine approach
ASIS: Anterior superior iliac spine
DAA: Direct anterior approach
IL: Inguinal ligament
LFCN: Lateral femoral cutaneous nerve
THA: Total hip arthroplasty
